# The application of virtual reality and augmented reality in Oral & Maxillofacial Surgery

**DOI:** 10.1186/s12903-019-0937-8

**Published:** 2019-11-08

**Authors:** Ashraf Ayoub, Yeshwanth Pulijala

**Affiliations:** Scottish Craniofacial Research Group, Glasgow University MVLS College, School of Medicine, Dentistry and Nursing, Glasgow University Dental School, 378 Sauchiehall Street, Glasgow, G2 3JZ UK

**Keywords:** Virtual reality, Augment reality, Haptic, Navigation, Oral surgery

## Abstract

**Background:**

Virtual reality is the science of creating a virtual environment for the assessment of various anatomical regions of the body for the diagnosis, planning and surgical training. Augmented reality is the superimposition of a 3D real environment specific to individual patient onto the surgical filed using semi-transparent glasses to augment the virtual scene.. The aim of this study is to provide an over view of the literature on the application of virtual and augmented reality in oral & maxillofacial surgery.

**Methods:**

We reviewed the literature and the existing database using Ovid MEDLINE search, Cochran Library and PubMed. All the studies in the English literature in the last 10 years, from 2009 to 2019 were included.

**Results:**

We identified 101 articles related the broad application of virtual reality in oral & maxillofacial surgery. These included the following: Eight systematic reviews, 4 expert reviews, 9 case reports, 5 retrospective surveys, 2 historical perspectives, 13 manuscripts on virtual education and training, 5 on haptic technology, 4 on augmented reality, 10 on image fusion, 41 articles on the prediction planning for orthognathic surgery and maxillofacial reconstruction. Dental implantology and orthognathic surgery are the most frequent applications of virtual reality and augmented reality. Virtual planning improved the accuracy of inserting dental implants using either a statistic guidance or dynamic navigation. In orthognathic surgery, prediction planning and intraoperative navigation are the main applications of virtual reality. Virtual reality has been utilised to improve the delivery of education and the quality of training in oral & maxillofacial surgery by creating a virtual environment of the surgical procedure. Haptic feedback provided an additional immersive reality to improve manual dexterity and improve clinical training.

**Conclusion:**

Virtual and augmented reality have contributed to the planning of maxillofacial procedures and surgery training. Few articles highlighted the importance of this technology in improving the quality of patients’ care. There are limited prospective randomized studies comparing the impact of virtual reality with the standard methods in delivering oral surgery education.

## Background

Virtual reality “near reality” is the art and science of creating a virtual environment that provides a standardized, safe and flexible platforms for the assessment of various anatomical regions of the body for examination, diagnosis, planning and for the surgical training. To achieve this objective the user of this technology should be exposed to a realistic multidimensional visual stimulus. This to allow the full integration of cognitive, motor and mental functions of the operator. So, virtual reality describes a 3D computer generated environment which can be readily explored and interacted with by a person [1].

Augmented reality combines virtual reality with a 3D real environment specific to individual patient via a sophisticated registration process to achieve an integral image which augments the virtual scene with the real one. The integrated image is superimposed on the real environment using semi-transparent glass [2].

Based on the level of presence experienced by a user, virtual reality technology can be broadly classified into immersive virtual reality and non-immersive virtual reality. The basic elements of immersive reality experience include interactivity and involvement of the user within the virtual environment to create a sense of being “present” in the environment. Immersive virtual reality combines virtual reality with the added characteristics of the captured environment to provide the operator the sense of being in the scene, able to visualise the recorded image in 3D, and interact using a sophisticate wearable device that detects eye movements and track leap motions of the hands. Non-immersive virtual reality involves computer generated experiences on a desktop, while the user interacts with a mouse, in a virtual environment. Conventional surgical simulations fall under this category [3].

The advances in computing power have made simulated images much more realistic and much faster to create. The concept of virtual reality requires the development of specialised software to manipulate the recorded 3D images of the dental and oro-facial morphology. Therefore, it is important to highlight the existing methods of recording the 3D dental, skeletal and soft tissue structures of the dentofacial anatomy and be cognisant of the strength and the limitation of each method.

Different techniques have been developed for capturing dental, facial soft tissue and hard tissue data to produce 3D virtual models for the analysis and surgical planning. These techniques helped to overcome the drawbacks of the 2D photographs and radiographs. Four main types of 3D imaging systems have been used to capture dental and oro-facial structures which include cone-beam computed tomography (CBCT) Laser scanner, structured light scanner, and stereophotogrammetry [4]. These are essential for virtual planning of the surgical correction of dento-facial deformities, maxillofacial reconstruction after cancer resection and the simulation of facial fractures. The 3D acquisition of the various tissues of the head and neck region provides a realistic platform for maxillofacial training. The recorded images can be superimposed into the patient, using semi-transparent glasses, to allow the surgical procedure to be carried out in environment of augmented virtual reality.

CBCT is a 3D radiographic imaging of the craniofacial region; it is also known as “digital volume topography”. Even though CBCT is excellent in the imaging of hard tissue, the soft tissues are of poor contrast and the method does not produce the normal photorealistic appearance and the texture of the skin of the face. The stereophotogrammety allows the 3D recording of facial texture which can be easily superimposed the 3D surface image of the CBCT. The time required for image acquisition is less than one millisecond, and it is highly accurate and reliable for the capture of face morphology. The capture 3D iumage of the skin can be accurately superimposed on the CBCT to produce a photorealisatic image of the faceover the captured facial skeleon [5].

Image artefacts are another limitation of CBCT, artefacts such as streaking, shading, and distortion are usually produced due to the presence of metallic restoration, fixed orthodontic appliances, or implants that are affecting the quality of the images. Therefore, the image of the defective dentition of the CBCT is usually replaced with the 3D image of the scanned dental models using either CT or laser scanner. The fusion of the images can be also achieved between the CBCT and the intra oral scans for orthognathic surgery planning, the accuracy of the method was within 0.5 mm [6].

### Aim of the study

Provide an over view of the literature on the application of virtual and augmented reality in oral & maxillofacial surgery.

## Methodology

We reviewed the literature and the existing database using Ovid MEDLINE search, Cochran Library and PubMed. All the studies in the English literature in the last 10 years, from 2009 to 2019, related to the application on virtual and or augmented reality in oral & maxillofacial surgery were considered. A set of key words guided the literature search including 3D, virtual reality, augmented reality, oral & maxillofacial surgery, dental and training. Key articles based on a robust methodology, adequate sample size and novel applications were retrieved for evaluation and the findings were presented in this manuscript.

Articles related to the detailed programming for virtual reality, abstracts, conference proceedings, letters to the editor, single case report, and those related to software development were excluded.

## Results

We identified 101 articles related the broad application of virtual reality in dentistry and oral & maxillofacial surgery. These were subdivided as follows; Eight systematic reviews [7–13], (Table 1), 4 expert reviews, 9 case reports, 5 retrospective surveys, 2 historical perspectives, 13 manuscripts on virtual education and training, 5 on haptic technology, 4 on augmented reality, 10 on image fusion, 41 articles on the prediction planning for orthognathic surgery and maxillofacial reconstruction. The results will be presented under two main categories, clinical applications and surgical training.

**Table 1:** **Table 1** Systematic reviews on the application of virual reality and augmented reality in oral and maxillofacial surgery

Authors	Year	Scope	Main findings	Conclusion	Recommendations
Joda et al [7]	2019	Augmented and virtual reality in dental medicine	The technologies were mainly used for educational training	Virtual reality is a promising tool that could deliver predictable outcomes	Establishing technological standards and developing approved applications are essential
Maliha et al. [8]	2018	Haptic, physical and web-based simulators in maxillary surgery	Most of the studies “10” were on virtual reality haptic based simulators.	no studies with high level of evidence	Studies on the effectiveness of the surgical simulators with high level of evidence are needed.
Huang Y et al [13]	2018	Application of virtual and augmented reality in dentistry	Computerized dental simulators provide the best training system for dental students	The methods are essential tools in the future of dental OSCE	The methods should be applied more extensively in medical treatment
Chen X, Hu J [9]	2018	Virtual reality based haptic simulation in oral surgery	Virtual and tactile fusion of virtual environment is effective for surgical simulation	Haptic technology provides excellent facility to improve surgical skills	Controlled studies are needed
Azarmehr et al	2017	Treatments and outcomes of surgical navigation	The accuracy of surgical navigation was within 1–2 mm	The method is useful addition to the surgical toolkit	Training is required due to the steep learning curve
Lin H, Lo J [10]	2015	Surgical simulation and intraoperative navigation in orthognathic surgery	The method provides precise translation of the surgical plan	Navigation facilitates intraoperative manipulation of the osteotomy segments	Randomised controlled trials are essential
Jayaratne et al [12]	2010	Computer assisted oral & maxillofacial surgery	Most focused on CBCT, stereophotogrammetry and surgical planning software	Accurate diagnosis can be achieved and can be executed via surgical navigation	Technology transfer from research to clinical care

### Clinical application

Technological advances in virtual and augmented reality are enabling the application of the methods in dentistry, oral and maxillofacial surgery is the primary area of application, dental implantology and orthognathic surgery are the most frequent application [14]. Most of the publications were on the assessment of the accuracy of virtual planning for orthognathic surgery [15]. Three-dimensional virtual surgery and mandibular reconstruction after cancer resection and reconstruction were the main applications of virtual reality [16]. Virtual planes for mandibular and maxillary reconstruction can be achieved with an excellent match. This was demonstrated on 30 cases of complex head and neck reconstruction including the planes of the resection, the length of the segmental defect and the distance between the transplanted segments and the remaining bone. There was excellent match between the virtual plans and the achieved results [17].

In a series of case reports the virtual surgical planning and hardware fabrication for open reduction and internal fixation of atrophic edentulous mandibular fractures were demonstrated [18–20].

In dental implantology, the accurate placement of dental implants is essential to meet the required functional and aesthetic demands [21]. Virtual reality has been extensively applied using the preoperative CBCT to determine the implant size, position, direction and proximity to vital structures. Various software packages are available for the virtual planning of dental implants [22]. The 3D virtual planning is then transferred onto the surgical field via either the static guide or the dynamic navigated approach [23].. The static transfer of the surgical plan is based on the virtual designing followed by fabrication of a surgical guide using computer-aided-design/computer-aided-manufacturing (CAD/CAM) to facilitate the insertion of dental implants. Various types of surgical guides are available based on the type of support, bony, mucosal or dental. A remarkable accuracy can be achieved with the sleeve-in-sleeve template is used in which multiple sleeves are applied and fixed to the surrounding bone to improve the precision of insertion of dental implants [24]. Various static guiding systems are available based on the CAD/CAM technology which includes EasyGuide, GPIS, Impla 3D, InVivoDental, Implant 3D, Nobel Bioguide and VIP (Implant Logic System) [25].

On the other hand, the dynamic navigation allows the real time adjustment of the direction of dental implant during surgery based on the virtual preoperative planning.

One of the main advantages of the dynamic navigation is the operator flexibility of changing the implant position to avoid compromised bony foundation and anatomical structures that may have not be detected during the presurgical planning phase. A high level of accuracy has been reported with the image guide implantology (IGI) system with an overall navigation error of 0.35 mm (and a mean angular deviation of less than 4 degrees [26]. However, it must be emphasised that the technology requires an expensive hard ware, significant learning curve and a rigorous intra-operative referencing and orientation process. Moreover, a disrupted surgical procedure may be encountered due to sensors being blocked during the navigation process.

No doubt, the virtual computerized implant dentistry has opened a new horizon in the management of complex cases where the anatomy of the jaw bones has been altered due to trauma or pathology. It improved the accuracy of implant placement where minimally invasive surgery is required in those who suffer from blood dsycrasias and radiation related bone damage.

Navigation within a virtual environment has been successfully used for the during orthognathic surgical [27], and for the repositioning of the maxilla to correct facial asymmetry [28]. The accuracy of the method was evaluated on 15 patients and ranged from 0.9 to 2 mm. An overview on the indication and the application of computer assisted navigation in oral and maxillofacial surgery was carried out on 104 cases, including 37 zygomatico-orbital maxillary fractures, 27 unilateral TMJ ankylosis, 29 craniofacial fibrous dysplasia, 9 mandibular hypertrophy, 3 bone tumours, two foreign body cases [29]. All the surgeries were performed under the guidance of the navigation system based on the preoperative simulation and superimposing the procedure in real time. The accuracy of the navigation system was assessed by measuring the discrepancies between the achieved results and the virtual plans. The mean error was 1.4 mm, it was concluded that navigation surgery is useful as it improved the accuracy of the performed procedure and reduced operation risks.

The application of augmented reality was mainly in dental implant placement and orthognathic surgery. A novel augmented reality system for displaying alveolar nerve bundles in maxillofacial surgery was recently developed. A novel approach based on fiducial markers within an occlusal splint was used to establish a relationship between the virtual image and the real object. The systems promise a broad clinical application [30]. The application of augmented reality system for oral and maxillofacial surgery was investigated [31]. The three-dimensional virtual image of osseous structures was projected into the patient’s body. This helped the surgeons to avoid important structure inside the bone during surgery. Surgical procedures including hole drilling, screw fixation were performed and guided by the augmented reality, the overall precision of the system was within 1 mm.

The application of augmented reality for dental implantology was recently tested in two cases [32]. The study explored the feasibility of a virtual display of the implant position, using specific glasses, on surgical field for surgical navigation in augmented reality. The two virtual environments did not affect the accuracy of the surgical procedure. However, this prove of consent study promises a wider application in maxillofacial surgery.

For an immersive virtual experience, the user wears a head-mounted displays or goggles to engage his visual senses, headphones to engage his auditory senses, and gloves to engage his tactile sense. Rapid advances in technology and research led to the introduction of commercially available high quality immersive virtual reality devices including Oculus Rift (Te 2015) [33], Google Daydream (Google 2017) [34], Gear VR (Samsung, 2015) [35], Goggle Cardboard (Goggle, 2015a) [36] and HTC Vive (Corp 2015) [37]. Among these Google Daydream, Gear VR and Google Cardboard headsets can create a portable virtual reality environment as they work with smartphones. These lead physicians to explore the potential of immersive spherical videos in medical education.

The addition of haptic technology which provides the operator with tactile feedback of the touched or held digital object on the computer screen, has augmented virtual reality and created a more realistic environment for clinical training. Most of the haptic technology applications in immersive virtual environment were carried out on experimental models [38]. A haptic-assisted craniomaxillofacial surgery planning system was applied for the restoration of skeletal anatomy in complex trauma cases **[**39]. A virtual model was derived from the patient CT data. The developed system combined stereo visualization with six degrees of freedom, high fidelity haptic feedback that enabled the analysis, planning, testing options for restoring bony segmental defects. The system has the potential to be a powerful tool in oral and maxillofacial surgical planning. The literature showed that most of the application of surgical navigation was in orthognathic surgery to improve the accuracy of guiding the osteotomy segment of the jaw bones according to the pre-planned position [11].

### Surgical training

Virtual reality has been utilised to improve the delivery of education and the quality of training in dentistry and in oral & maxillofacial surgery [40]. Voxel Man Simulator was used for virtual apicoectomy procedure and found that out of 53 dental students who undertook virtual apicoectomy, 51 were positive regarding the impact of virtual simulation as an additional modality in dental education. The trainees indicated that the integrated force feedback (e.g. simulation of haptic pressure), spatial 3D perception, and image resolution of the simulator were key features for virtual training of the dental surgical procedures. Trainees also developed the ability to self-assess their performance which is a valuable skill in surgery which is essential for the refinement of surgical technique. This study also proposed that application of virtual surgery using the 3D reconstruction of patient’s anatomy might help surgeons to plan complex surgical procedures [41].

Recently, the impact of virtual reality as a training tool for surgical procedures was evaluated in a cross-sectional study to validate a novel virtual simulator for orbital reconstruction, and a training tool in oral and maxillofacial surgery [42]. A novel virtual reality approach based on haptic technology was introduced and validated for computer aided cephalometry. Twenty-one dental surgeons performed a range of case studies using haptic-enabled digital cephalometric analysis. They proved that by providing a sense of touch the errors in cephalometric analysis has been reduced and the landmarking became more feasible and more intuitive [43].

**Fig. 1:**
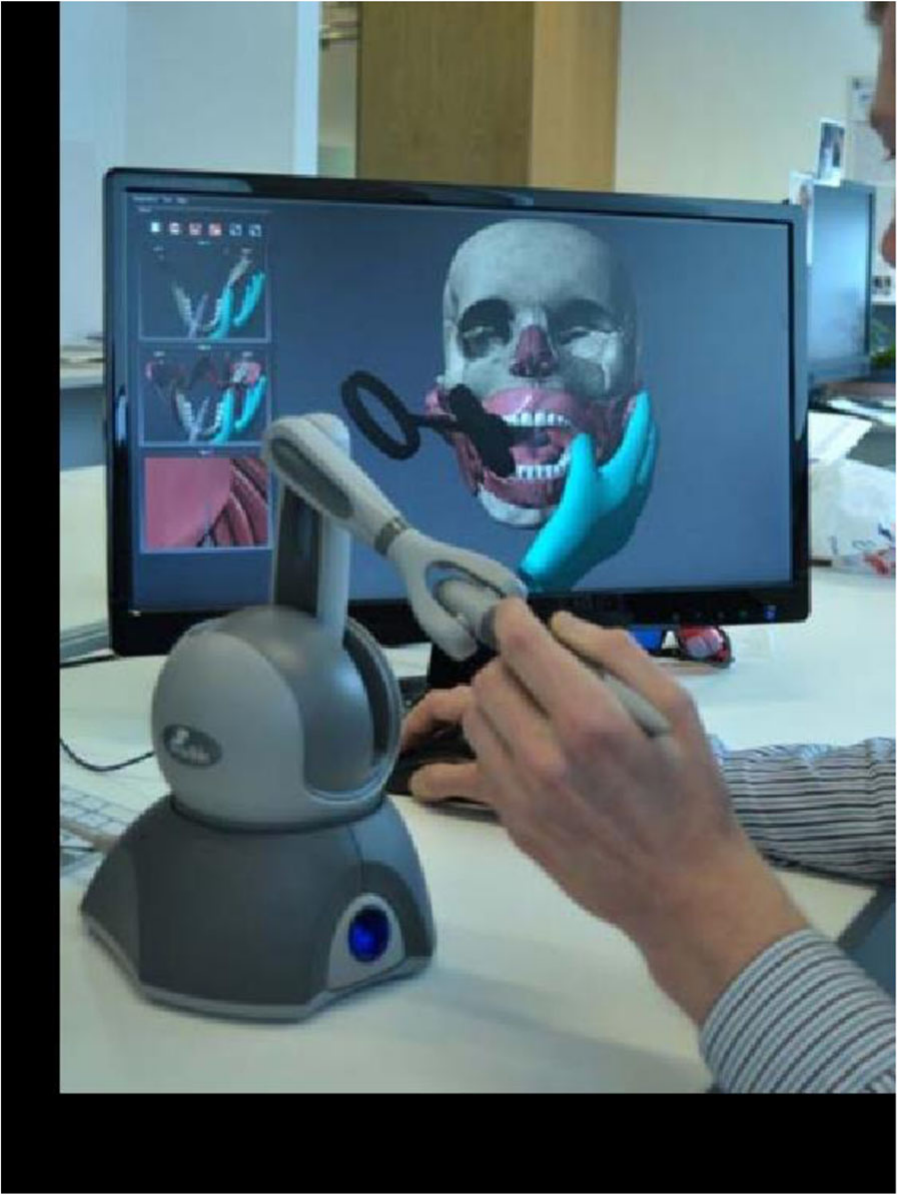
Demonstration of haptic technology of injection of the inferior dental nerve (taken from Anderson, P., Chapman, P., Ma, M. and Rea, P. (2013) Real-time medical visualization of human head and neck anatomy and its applications for dental training and simulation. Current Medical Imaging Reviews, 9(4), pp. 298–308

The applicability of using 3D visualisation in dental training was also reported where a haptic dental injection was developed for inferior alveolar nerve block injection as shown in Fig. 1, they also developed a virtual training system (VR-MFS) with advanced haptic feedback and immersive workbench [44]. In addition to drilling, this system allowed cutting and milling aspects of the bones. 3D stereoscopic visualisation on an immersive workbench provided visual, tactile and aural feedback bringing it close to reality. Le Fort 1 maxillary surgery was simulated in this system; the cutting and drilling trajectories and were compared with a preoperative plan for evaluation. The study found that expert surgeons’ trajectories were close to the plan when compared to the novices. Though the experts believed that VR-MFS could be used for skill development, they pointed out that the system lacked realistic simulation that is required for effective training.

The implementation of web-based virtual patient simulation program to teach dental students oral surgery has been investigated. Virtual reality has improved students’ knowledge and proved to be effective in teaching clinical reasoning and patient evaluation [45].

Recently, the use and the clinical application of virtual reality in pre-clinical dental education was reviewed. Four educational thematic areas were identified which included the simulation hardware, the realism of virtual simulation, scoring system for the assessment of virtual reality and the validation of the emerged systems. Four types of simulators have been used for dental education which included desktop pcs, haptic desktops, and dental skill trainers and digitally enhanced phantom heads. It was clear there were no established educational standards for dental simulators. Most of the available dental simulators have not been validated [46].

**Fig. 2:**
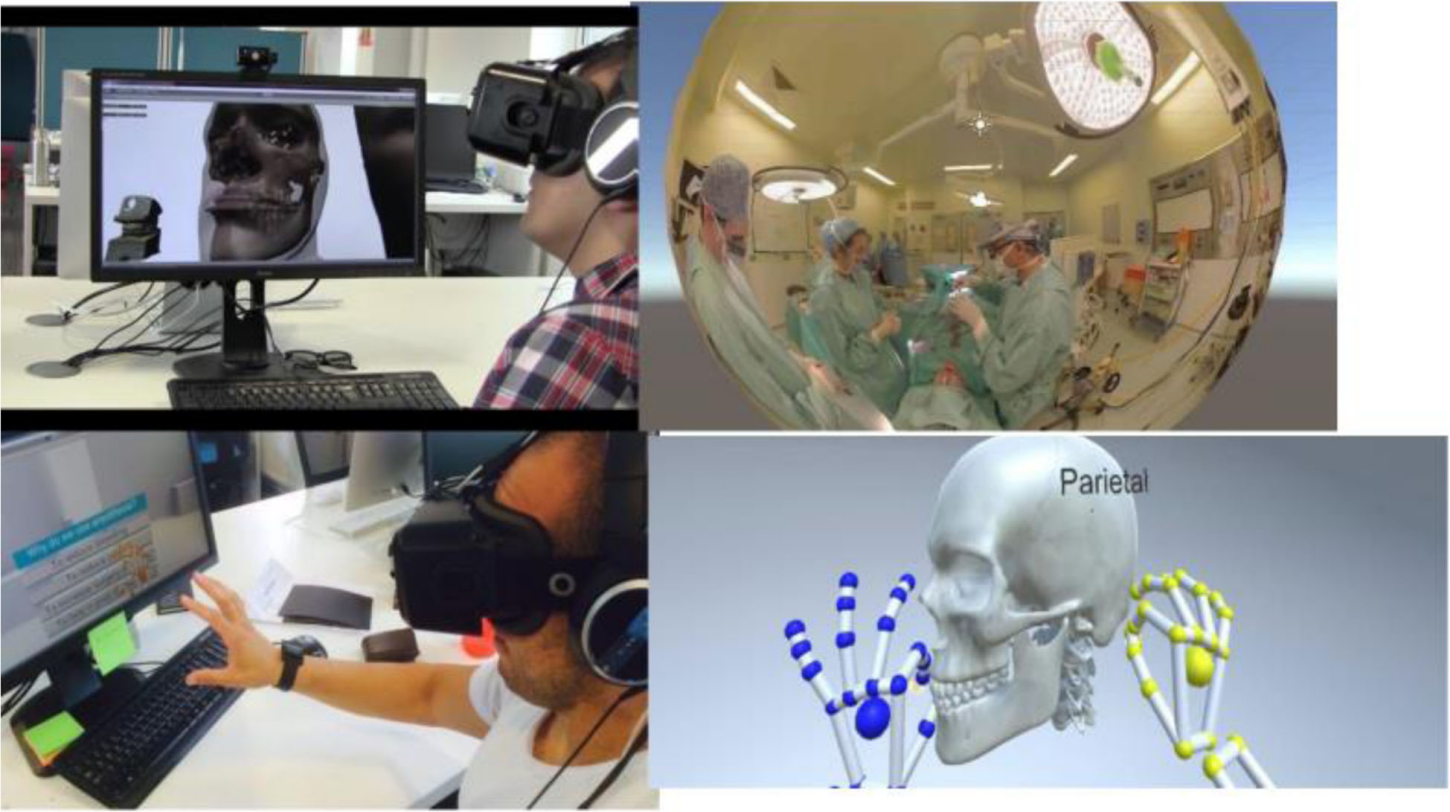
Oculus Rift showing 3D digital data, the operating theatre, the leap motion sensor tracks the trainee’s hands to select items from the menu or apply a surgical instrument (PhD thesis of Yeshwanth Pulijala The University of Huddersfield, 2017)

On the other hand, a stereoscopic 3D videos using immersive reality was developed (Fig. 2) and its impact on improving the non-surgical skills among trainees was investigated [47]. Based on the 3D computer-generated model of the operating room the trainees can navigate, explore and interact with the digital images of the patient’s data. A Leap Motion sensor tracks the trainee’s hands (Fig. 2) to provide a multi-sensory interactive learning experience. The users were able to choose a specific application and zoom in on certain items within a surgical menu. Through specific gestures the trainees can interact with the anatomy of the maxillofacial region and select the most appropriate surgical instrument to perform certain surgical procedure. The developed programme tests the trainees’ knowledge through a quiz scene. The efficacy of VR Surgery in training novices was assessed. A single-blind prospective randomised controlled trial confirmed that the group of the trainees who used VR Surgery performed better than the control group.

Virtual reality has been utilised to create a learning environment for training in maxillofacial emergencies to improve knowledge and confidence of junior trainees [48]. The pilot studies showed improved in the two examined domains, further was recommended by the investigators. Following the same theme of virtual surgical simulation, the feasibility of tree-structure architectonic model to simplify virtual orthognathic surgical was explored [49]. This was tested on a group of patients who require orthognathic surgery. The operators were immersed in the virtual environment and tactile feedback was perceived which augmented the training opportunities [49].

The importance of virtual reality in standardizing clinical education to facilitate learning and practicing has been highlighted. The methods encouraged the students to learn by themselves which can reduce faculty time significantly. CDS-100 simulator, designed by EPED Inc. has been shown to be effective computerised tool as it provided 3D real time accurate feedback for endodontic and prosthetic applications. The objective structured clinical examination (OSCE) can be easily incorporated. The authors highlighted the importance of the real time navigation technology in dentistry and emphasised the need for high quality medical images for accurate implementation of the technology [13].

It has been recently highlighted that the current customized augmented reality systems have not been fully validated by independent teams, they provide good results in simple experimental models. The superimposition of digital images is easier on bony structures, therefore, the application of this innovation in oral & maxillofacial surgery is readily achievable and prepare the way for a wider application [50].

## Conclusion

In conclusion, virtual reality and augmented reality have contributed to the surgical practice and training in oral & maxillofacial surgery. Few articles highlighted the importance of this imaging innovation in improving the quality of care delivered to patients. The main application of virtual reality is in implantology and orthognathic surgical. Virtual reality facilitated the restoration of orbital floor following blow out fracture and the planning of mandibular reconstruction following cancer resection. There are limited prospective randomized studies to assess the impact of virtual reality with the standard methods of delivering education or carrying out oral surgical procedures. Most of the existing models of simulation focused on the technical skills of the surgical trainees. Non-technical skills including cognitive development, interpersonal communication, teamwork, and emergency management are hardly touched upon except in few studies. The technical skills learnt by the trainees on the virtual surgery simulators are limited but expected to transfer into a stressful environment of operating theatre. However, as a surgical procedure is a combination of expert anatomical knowledge, spatial visualisation, judgment and inter-professional teamwork, it is essential to give a holistic learning experience to the trainees. Hence, there is a gap in the modern simulators developed for dentistry and oral and maxillofacial surgery, which needs to be met adequately. Researchers attempted the use of serious games and gamification of simulations to overcome these training obstacles. Further studies are required to compare the impact of augmented reality in improving the quality of care delivered to patients with the standard approaches.

## Data Availability

Existing literature in Ovid & PubMed. The data sheets generated during the current study area available by contacting the first author (AA) via his email address.
